# Perimeter Security Utilizing Thermal Object Detection

**DOI:** 10.3390/s25216680

**Published:** 2025-11-01

**Authors:** Georgios Orfanidis, Konstantinos Ioannidis, Stefanos Vrochidis, Ioannis Kompatsiaris

**Affiliations:** Centre for Research and Technology Hellas—CERTH, Thermi, GR 57001 Thessaloniki, Greece

**Keywords:** critical infrastructure, object detection, surveillance, thermal, Infrared

## Abstract

**Highlights:**

**What are the main findings?**

- Thermal object detection increases surveillance capabilities.
- Deploying a thermal surveillance system is a practical and economical solution.

**What is the implication of the main finding?**

- Thermal detection can greatly complement other surveillance systems.
- More thermal detection systems can be utilized.

**Abstract:**

In recent years, an increasing interest in artificial intelligence applications in a widespread spectrum of fields which include, among others, robotics, communications, artistic creations, security and protection technologies, etc., has been observed. Of the latter categories, one field which has largely benefitted is surveillance and security technologies. This fact is combined with an increase in omnipresent automatic surveillance system installations which pave the way to new technologies. Technologies that are being promoted are the ones offering uninterrupted, robust, efficient and reliable operation. In this work, we examine the ability of thermal automatic detection systems to fulfill their role as an essential part of such a mechanism. The primary advantage of thermal detection is the potential to provide a 24-h uninterrupted detection service exploiting its innate robustness against environmental or weather changes and shifts in illumination conditions. For providing a reliable security mechanism, a second requirement is considered sine qua non: the efficiency of the system in order to provide timely alerts for potential threats and incidents. In this work, we evaluate various efficient object detection models operating solely in the thermal/infrared spectrum to examine their role as potential backbone detectors in surveillance systems.

## 1. Introduction

Object detection is one of the fundamental tasks in computer vision. Nevertheless, the introduction of deep learning techniques and more specifically Convolutional Neural Networks in recent years has leveraged the performance of the relevant models to unprecedented levels. The latter fact has permitted the introduction of detection techniques in various automated solutions for surveillance and security purposes. Additionally, although the primary field to apply such monitoring systems is the visual spectrum (RGB), there are multiple cases where this obvious selection proved insufficient. Detection systems operating in the visual spectrum have reached impressive levels of performance since the introduction of the early stages of deep learning techniques. Pioneering works include Faster RCNN [1], Single Shot Detector (SSD) [2]—an innovative single-step network, and You Only Look Once (Yolo)—an efficient oriented model [3]. Those works are based on Convolutional Neural Networks which tend to dominate the detection field. Nevertheless, other approaches also exist which utilize different architectures like Transformer. Carion in [4] provided end-to-end architecture to train a transformer and provide a detection model. In general, the works are focused on improving the detection ability while simultaneously providing swift models with increased usability.

In fair weather and illumination conditions, the performance of those detection systems is unparalleled [5]. On the other hand, when the conditions are not thus favorable the detection ability diminishes significantly. At those conditions a complementary system is useful to provide detections. Missing objects due to low visibility is an unacceptable case for robust protection systems and this is when thermal object detection is utilized. The latter provides decent detecting capability in harsh weather and illumination conditions. To further elaborate, harsh conditions might include foggy, misty or rainy weather, low visibility might also include dawn, sunset or heavily cloudy weather, etc. [5]. In practice thermal objects can operate under a large range of climatic conditions, including fair, cloudy, dawn, misty, rainy and foggy weather with minimal impact on its detection ability. This feature is unique compared to other visual detections. Additionally, specifically the thermal cameras can operate without any external "light" source by detecting the thermal radiation of each object, which provides an affordable alternative to the installation of multiple light sources to illuminate the whole area around the monitored area [5]. Finally, another advantage of thermal detection is the smaller image resolution of the cameras along with the single-channel processing which can boost the processing time of the system.

Due to the increased interest of the community, a large number of datasets specifically created have been published and utilized to promote the research on the generic object detection field. This has aimed at the comparison of works and the progress in the field. Datasets such as Common objects in context (Coco) [6] operate as benchmark datasets for this purpose. On the thermal spectrum, unfortunately, there is not any massive dataset which could uptake this role. In a study on the datasets being utilized in thermal detections, almost three out of four works were reporting results in non-publicly available datasets [7]. 

For the purpose of this work, public datasets were utilized, which included thermal images as the intention is to validate the credibility of thermal detectors as security tools. In order to provide an easily maintainable and constructible application, the focus was only on public datasets. Some of these datasets also contained visual images but only the thermal part of the dataset was utilized, as is the case of FLIR-ADAS [8]. A certain issue is the availability of objects of interest inside the public datasets. Within the aspect of this work, only those relevant to the surveillance purposes were kept and all others were discarded. Similarly, we have annotated relevant objects in cases these were not available. The relevant classes included persons, cars, trucks, buses, motorcycles, bicycles and fires. The person class is essential for every surveillance task as it is obvious. The next five classes include the most commonly utilized vehicles and thus, they were considered indicative of human presence and activity in the surveyed area. For this reason, they were also included in the relevant detectable classes. Finally, instances of fire constitute one of the major threats for infrastructures, installations, building and persons, and thus, it was incorporated to the final classes of interest. Every instance of those objects was annotated in the dataset being utilized.

Additionally, three different detection models were examined for the surveillance task. Two of them were of the Yolo family, Yolov8 [9] and Yolov11 [10] while the third one was from the transformer doctrine but specifically focusing on efficiency Real Time Detr (RT-Detr) [11]. The inclusion of three models permitted the research into the best performing model under different parameters.

The main contributions of this paper are the research of the performance of efficient real-time object detectors in the IR/thermal spectrum, in varying parameters and model sizes. Additionally, the main question that this work attempts to answer is whether the existing detection model can operate in a robust and reliable way in order to constitute the backbone of a 24/7 thermal surveillance detection system.

This paper is organized as follows: an overview of related work is presented in the following section. A small discussion of the unique features of IR/thermal radiation is presented in Section 3. Section 4 discusses the datasets being utilized for this work, while the next section aggregates the parameters being applied, the actual results and their impact in the model selection. Finally, the last section, Section 6, concludes the paper and summarizes the main ideas deducted from it.

## 2. Related Work

This work utilizes a deep learning approach for detection purposes but deploys its algorithm solely on a thermal bandwidth, and thus, this section will summarize related works in both of these fields. As mentioned in the Introduction, the emergence of deep learning in the detection field has created many remarkable works. Initial approaches focused on achieving better results but as the advancements accumulated, a large portion of the innovative works shifted their focus on promoting efficient yet effective models. A major chapter on these efficient models is the Yolo tradition. This tradition has created a whole series of detectors [12] which have as their primary focus to provide a model which is as efficient as possible. Each subsequent Yolo version attempts to include some innovation in the architecture while maintaining its fast-processing ability [13].

The initial works were also almost exclusively based on Convolutional Neural Networks. Further implementations introduced newer architecture like visual transformers and a discussion commenced [14]. Nowadays the best performance is achieved by visual transformers, yet they typically require much data, are slower and more resource-consuming than their counterpart. Examples are implementations like Co-Detr [15], which, although it achieves state-of-the-art performance in datasets like Coco, failed to provide a lightweight efficient model. A new generation of transformers attempted to crack into the efficient models based on CNN, employing efficient alternatives to achieve real-time processing like the aforementioned RT-Detr.

Regarding the other field of interest of this paper, thermal object detection, the initial interest of the community focused primarily or even exclusively on human detection. Although the reason for focusing primarily or even exclusively on person monitoring might be traced back to the pre-deep learning era where the inclusion of many classes of interest was quite a hard task to accomplish, there are also other practical causes. The human presence per se is crucial and could provide valuable information and increase the environmental awareness of the system. Nevertheless, detecting more classes is typically more useful and can leverage the practical uses of the solution. A typical example is [16] where the authors presented a system for thermal detection of persons and vehicles. This work is pre-deep learning and thus, it utilizes machine learning techniques. Another attempt at thermal detection is presented in [17], where the authors utilize a multi-camera surveillance system based on the Yolov4 [18] model to monitor crowds in indoor places. 

The authors of [5] created their own dataset to examine the thermal detection in unfavorable weather conditions. The dataset included frames captured in clear, foggy and rainy weather and primarily focused on detected persons in various positions (there was a human vs. non-human—dog—task included also), while the detectors where all CNN-based models. An example of multi-person localization via the use of a Thermopile sensor is presented in [19], comparing Yolov5 [20] and Detr [5] algorithms. The results are anonymous by nature due to the low resolution of the sensor, but on the other hand, they lack any clarity for visualization purposes.

In another work, [21], the authors utilized SSD with mobileNet v1 and v2 to detect three classes, car, bicycle, and person, on FLIR-ADAS dataset v1. The lack of IR/thermal extended datasets resulted in attempts to transfer features from the richer domain, such as the visual one, in works like [22], where pseudo-multimodal pairs were generated in order to improve performance. Researchers often attempt to improve the performance of the developed applications, especially to achieve real-time performance, as in [23], where the authors focused on vehicles’ detection in Infrared images utilizing a modified Yolov7 model [24].

One popular application for thermal object detection is Autonomous Driving. In [25], the authors introduced a new VGG-based detector named TIRNet and focused on providing an efficient system which was evaluated on a custom-compiled dataset named CTIR and on a KAIST dataset [26]. The dataset availability for the thermal spectrum is apparent and in this field. For example, a new interesting visual dataset appears in [27], which attempts to cover more diverse sceneries. One approach for taking advantage of these datasets is to transfer knowledge from the visual to the thermal spectrum. For example, in another work which also focuses on Autonomous Driving [28], it uses transfer learning to provide low-level feature transfer from the visual to the thermal domain in order to improve the performance in low lighting conditions. The models used were SSD and Faster RCNN. Increasing environmental awareness is crucial for Autonomous Driving and the authors in [29] attempted in an innovative manner to enrich this knowledge by detecting roadside wild animals utilizing thermal imagery and a Yolov8 variation. This approach aims at employment in embedded systems in order to mitigate the chance of animal–vehicle collision.

As stated before, the typical resolution of thermal imagery is small and thus, deployment of thermal object detection models in embedded devices might seem reasonable. Nevertheless, the detection of small objects in the latter already stretched resolution is challenging, especially when combined with the innate scarcity of detailed textures for thermal imagery. Applications which attempt to contribute to this task are always interesting such as the case in [30], where the authors utilize a Yolov5 [20] -based model deployed in integrated circuits TIFAD [31]. The application datasets were mainly TIFAD [32], and for model robustness, evaluation HRSID [33]. The relevant works including thermal object detection on embedded devices are even more limited and the ones published attempt to fill in the gap in this research field by introducing model improvements, as in the case of [34] for a variation in Yolov3 [35] but the use of non-benchmark datasets ultimately restricts the impact on the community.

Another application of thermal detection is for research and rescue operations, especially during nighttime. One such example is presented in [36], where the authors employed a thermal object detection mechanism to detect waterborne individuals at nighttime when it is more challenging to accomplish. They also compiled a custom dataset to train and evaluate their model which was based on Yolov5 architecture [20].

As mentioned before, in the surveillance thermal object detection subtask, works often focus on person detection, like in [37], where the authors present a method for background removal and which is applied on two sequences of the CDNet-2014 dataset [38]. Depending on the problem to be solved, the classes of interest might include more objects, like in [39], which attempts to detect animals in thermal imagery as it considers them a threat for crops and farmers especially during the night. Due to object instance sparsity, a GAN, more specifically ThermalGAN [40], was utilized to increase the number of training samples while the selected model was Yolov4. Other works attempt to unify the visual and thermal detection subtasks, considering they are complementary to each other. In [41], the three classes were detected animals, people and vehicles, while the model being utilized was Yolov3 [35] and the focus was the development of an outdoor surveillance system. A related task for mitigating potential threats was detecting UAVs utilizing thermal imagery [42]. The authors in the latter publication evaluated four models, more specifically Yolov9 [43], GELAN [44], DETR and ViTDet [45], in the anti-UAV Dataset compiled during the 2023 CVPR [46]. The best performance (in mAP) was achieved by GELAN while the faster detector was Yolov9.

As is obvious in the literature, thermal object detection field is deprived of the rich, extended benchmark datasets available in visual object detection. On the other hand, shutting down the entire department of thermal detections deprives any system of the advantages of the thermal spectrum. Thus, many researchers focused on leveraging both spectrums to their advantage. One such work, which aims at providing a lightweight yet effective multi-modal fusion model is [47]. The main mechanism utilized contains channel switching and spatial attention (CSSA), while the evaluation was performed in FLIR and LLVIP [48] datasets. Another multimodal system [49] performs late fusion to the detections from different modalities while applying a simple probabilistic ensembling approach to enhance the scores of consensual detections. Due to its late fusion nature, it can be applied to both aligned as well unaligned multimodal datasets. An interesting network which further expands the multimodality and encompasses three modalities in a Confluent Triple-Flow Network is presented in [50]. The focus of the paper was to locate salient objects by utilizing three flows: a visual, a thermal and a complementary one containing information from both latter flows utilizing a divide-and-conquer approach to independently process each flow while simultaneously extracting complementary features. Finally, they employ their own compiled dataset VT-IMAG to evaluate the performance of their mechanism.

## 3. IR-Thermal Radiation

Infrared (IR and sometimes also called infrared light) is an electromagnetic radiation (EMR) which has wavelengths longer than visible light but shorter than microwaves. Its name denotes that it is under (infra) the red band of the visual spectrum which is the nearest to IR. There is no strict definition of the boundaries of the IR band, but it is roughly between 780 nm and 1 mm wavelengths. It is not visible by the human eye, yet it transfers or reflects heat. The IR band is divided into an active IR band and a thermal (passive) IR band [51]. The active band covers the part near the visual spectrum (0.7-2.5μm) and it is divided into the NIR (near-infrared) and the SWIR (shortwave infrared) spectrum. The SWIR has slightly better performance when low levels of obscurants like fog and smoke are present. The passive IR band on the other hand covers spectra most distant to the visual band. It is divided into the Mid-Wave (MWIR) and the Long-Wave InfraRed (LWIR) band. Both bands are capable of capturing the emission of heat radiation from monitored subjects, but MWIR also has some reflective properties, whereas LWIR is comprised almost entirely of emitted radiation [51]. In practice, the difference between the active (reflected IR radiation) and passive (thermal IR radiation) is the ability of the latter to operate without the requirement of an external source of light or heat (which would be reflected by the object’s surface) and thus, is highly robust to illumination fluctuations and weather conditions, while it can be fully functional in the darkness [52]. The authors in [53] employ three modalities—more specifically, visual, thermal and Lidar input, to perform 3D object detection and eliminate blind spots for navigation purposes. The detector employs a Swin Transformer [54] to cope with the detection part.

## 4. Datasets

As mentioned before for IR/thermal object detection, the available public datasets are quite limited compared to their counterpart visual object detection datasets. In [7] where thorough research has been performed on the availability of such datasets, from a reported 27 works on the field, 18 use a self-compiled dataset for their research and 2 did not report their dataset. In summary, 20 out of 27 works (over 74%) did not utilize any public dataset. This fact proves the lack of a universally accepted, well-established public dataset which would operate as a benchmark for result reporting as is the case for Coco for visual object detection. As is obvious, this is a huge disadvantage for comparing works and promoting innovation in the field. Possibly, the best-known datasets in this field are KAIST and FLIR-ADAS multispectral datasets. KAIST contains over 95k color-thermal image pairs with 640 × 480 resolution but only three classes involving people are annotated: person, people, and cyclist. All other objects are not annotated, and in order to utilize this dataset, a re-annotation is required. 

For the purposes of this work, four different datasets were utilized: FLIR-ADAS, HIT-UAV [55], Corsican Fire Dataset [56] and Flame Dataset [57]. As mentioned before, we have included only the objects which are relevant to the surveillance task and ignored all other objects. Additionally, we have thoroughly examined the images provided to annotate object instances we are interested in but that were not originally annotated (as, for example, fire instances).

FLIR-ADAS on the other hand provides annotations for 16 classes of interest: 15 specific ones and 1 collective class for the remaining objects not belonging to any other class. More specifically, the classes are Person, Bike, Car, Motorcycle, Bus, Train, Truck, Traffic light, Fire Hydrant, Street Sign, Dog, Skateboard, Stroller, Scooter and Other Vehicle. Of those, the first 5 and Truck were included in the final dataset being utilized in this work. The perspective under which the images were captured is from a moving car since the dataset’s initial purpose is for Autonomous Driving.

HIT-UAV is a high-altitude thermal dataset targeted at object detection. It comprises 2898 thermal images captured from the UAV perspective in various scenarios, such as schools, parking lots, roads, and playgrounds. It contains 5 classes: 3 specific ones, 1 collective for the remaining vehicles and one labeled Don’t Care. The classes are Car, Person, Bicycle, Other Vehicle and Don’t Care. For the purpose of the surveillance task, the first 3 classes were selected. 

In Table 1, the exact classes used from each dataset along with the actual object instances included in the dataset are presented. The last column provided information for the accumulated dataset which was utilized for the training of the models. As can be observed, there are 7 classes included in the final compiled version. Nevertheless, the dataset is quite unbalanced, with 2 classes dominating the dataset, Person and Car, and accounting for nearly 90% of all instances. The latter distribution has an impact on the ability of the model to predict certain classes. 

The Corsican Fire Dataset is a dataset which focuses on fire detection. It contains both visual and near-infrared images and also provides segmentation masks. If focuses on outdoor fire instances and aims at providing a fire evolving dataset to promote scientific research in the field. In total, 640 infrared images were obtained to be included in the experiments conducted in this paper. It is worth noting that the dataset contains a little more visual than infrared images. 

The Flame Dataset is a multi-spectral dataset which focuses on external fire detection. It contains both visual and thermal videos of fires. We have selected a video of the collection which contains thermal video footage, extracted the images every 180 frames and manually annotated the Fire and Person instances in the frames. A total of 742 frames were acquired, of which 581 frames contained Fire instances and were kept in the dataset.

The type of classes we chose the model to be trained on are based on the ones which reveal human presence or activity [58]. For thermal object detection, these include human figures and various vehicle types (Car, Truck, Bus, Motorcycle, Bicycle). Additionally, as stated, from the natural disasters list [58], we have also included fire instances as a crucial yet detectable threat which can expand the system’s practical use. 

The four datasets present different qualities and offer a more complete figure of the detecting ability for the models under examination. The larger number of instances is contained in the FLIR-ADAS dataset by a large margin. This dataset is the most challenging one, as can be seen by comparing the results in the three result Tables. As regards the dataset split, when an official data split was available, it was utilized in the experiments. This was the case for the FLIR-ADAS and HIT-UAV datasets. Regarding the Flame v1 dataset, the adopted methodology involved splitting the dataset in a 90%-10% training–validation set but keeping the first roughly 10% of the video to minimize the dependency of the two sets. Obviously, there is a connection between the two sets, but it seems to be a safer choice for evaluating the performance of the model. Finally, for the Corsican Fire, a random 90%-10% split was also applied to create the two sets.

## 5. Results

In this section, we summarize the experiments and the results deduced by them. In the context of this work, an extended series of experiments have been conducted in order to evaluate the performance of efficient state-of-the-art object detection models and more specifically Yolov8, Yolov11 and RT-DETR v2 while being operated in the IR/thermal spectrum. The experiments included both effectiveness as well as efficiency metrics. This way, a more comprehensive view of the models under inspection is provided and a safer conclusion can be drawn.

The lack of a large benchmark dataset has limited the options for providing results easily comparable to other works. For this reason, we opted for a compiled dataset from four distinct public datasets. We have included the results in each dataset separately along with the results in the compiled dataset. This way, it is easier to evaluate the performance of each model for the various parameters being utilized in each case. Although all datasets contain images in the IR spectrum, they exhibit different behavior and do not coincide in their best performance for the same set of parameters.

### 5.1. Training Parameters and Process

The parameters utilized while conducting the experiments remained the default ones, if not stated otherwise. More specifically, Yolov8 and Yolov11 shared the same parameters which include Adam for the first 10,000 iterations and SGD [59] with momentum for the rest of the iterations as the optimizer with a base-learning rate set to 0.01, a base weight decay equal to 0.0005, a momentum equal to 0.937, a linear learning rate schedule, certain warm-up iterations and an exponential moving average (EMA) decay set to 0.9999.

RT-Detr v2, on the other hand, utilizes Res-Net [60] as its backbone which is pretrained in ImageNet [61]. Its optimizer is AdamW [62] with a fixed batch size of 3 (which provided the best results) and an EMA is applied with ema_decay = 0.9999. For the optional discrete sampling, an initial pre-training 6× with the grid_sample operator is applied followed by fine-tuning 1× with the discrete_sample operator. For scale-adaptive parameters, the learning rate is set to 1 × 10^−4^ and 5 × 10^−5^ for the backbones (ResNet18 and ResNet34, respectively) while for the detection branch, it is set for both models to 1 × 10^−4^.

Regarding the augmentation applied to the training phase, both models elaborated extensive augmentation techniques in order to improve the models’ robustness and generalization ability. On the one hand, Yolov8 and Yolov11 shared the same techniques which included color space alteration as well as geometric ones. Color space augmentation included random values in the HSV channels of the image and color swaps in the RGB space while the geometric ones refer to random rotation (by 90 degrees to fit the image in its rectangular initial shape), translation of the whole image, scaling of the image (either zooming in or out), shear translation, perspective transformation, flip up-down and left-right, a mosaic new image compiled of four independent images, a mixup of two images (a blending of two images onto each other) and finally a compilation of a cut corner of an image and the replacement of this part from a different image. On the other hand, RT-Detr v2 utilizes the random erasing of a part of the image, random lighting of the image, applying random distortion including hue, saturation, contrast, brightness and color channel swap, resizing the image, randomly adding canvas around the image, random cropping, randomly cutting a portion of an image and replacing it with that of another image, applying the blending of two images, random shifting in an image and finally a mosaic of four images. The most interesting part is that RT-Detr v2 applies a dynamic augmentation approach for the training, which means it switches off some of the augmentations for the final epochs of training.

Yolov8 and Yolov11 were trained for 100 epochs in each experiment, while their counterpart RT-Detr v2 was trained for 120 epochs following the setup provided by the developers of the respective models. The batch size for each experiment was set to the maximum allowed value for Yolos while for the transformer after experimentation it was kept fixed to three because the best performance was acquired using this parameter. The image resolution acquired four distinct values (as presented in the results Tables) utilized for both training and evaluation which are 640, 768, 896 and 1024. The model variation being chosen represents variations which typically present detection ability while maintaining their resources requirement to modest levels. As has been confirmed by the results, effectiveness of the models typically diminishes after a certain input image size. The training and evaluation were conducted in GeForce RTX 3080 (10 GB) and NVIDIA RTX 4090 (24 GB). The efficiency evaluation was specifically measured in the less efficient card (RTX 3080) to obtain a minimal efficiency metric.

### 5.2. Models’ Efficiency

The efficiency results are presented in Table 2, where it can be seen how each model performed in this field in regards to other parameters like image resolution and model variation. By simply comparing the speed of the models in inference mode, the faster one was Yolov8, followed by RT-Detr v2, and Yolov11 being the least efficient. The evaluation was performed on a modest GPU card, a GeForce RTX 3080, and it must be noted that all architectures achieved real-time performances (above 25 fps) for the models examined. Training was performed on a more powerful machine possessing a GeForce RTX 3090 card. More specifically, the models chosen to participate in these evaluation tests were the most efficient ones with the exception of nano variations which were skipped for both Yolov8 and Yolv11. Thus, the variations which participated in the experiments were the small and medium ones for the Yolo candidates and r18vd and r34vd for the Transformer candidate. Although the best single processing time is achieved by Yolov8, this trend does not seem to be consistent. On the contrary, the analysis of the overall performance of each architecture reveals a more complex pattern for the speed of each model. As already mentioned, the faster model is Yolov8, yet its efficiency is highly impacted by the image resolution. For example, in FLIR-ADAS, when utilizing the small variation, it achieves a fps 80 for the smallest resolution, 640 × 640, but drops to 42 for the largest, 1024 × 1024. This is a reduction of 47.5% which seems impressive. On the other hand, RT-DETRv2 achieves a speed of 73 fps for the 640 × 640 resolution but achieves 54 fps for the 1024 × 1024 input images (a reduction of 26.0%), with the latter performance being much higher than Yolov8’s counterpart. Yolov11, which shares a lot of components with the Yolov8 architecture, operates in a similar fashion and presents a drop of 46.4% (from 56 to 30 fps) between the smallest and largest image resolution inputs. Thus, the conclusion is that the efficiency of each model highly depends on the image resolution and the architecture used.

Another interesting fact is the way each model is affected by the increase in the image resolution. Yolos exhibit similar patterns regarding their performance, as greater images are being utilized: their processing speed decreases relatively quickly. The fastest model is Yolov8 for the small variation and 640 image resolution, but when the image utilized is increased to 1024 pixels, the speed drops to almost half: from 80 to 42 fps. Yolov11 is the least efficient model, with the best processing time at 56 fps for the smaller model in the 640-pixel analysis, which drops to 35 when 1024-pixel images are inserted in the system. This trend is even increased if the more powerful variation is utilized, the medium one. In this case, the speed varies from 56 to 30 fps for Yolov8 and 48 to 25 fps for the Yolov11 model. On the contrary, RT-Detr v2, while being the second fastest model with 73 fps in the smallest resolution (640 × 640), it scales more smoothly when 1024-pixel images are inserted in the model which can be processed at 54 fps (almost as the fastest performance of Yolov11). So, as a conclusion, RT-Detr can maintain its processing time more stably as the input image fluctuates, which is a huge advantage over Yolos.

### 5.3. Model’s Effectiveness

The results regarding the performance of each model for the various image resolutions are presented in Table 3. As can be seen from Table 2, the goal of providing real-time performance is matched for all models examined, but with big differences between model performances. Thus, this factor should be taken into consideration when impacting performance. Regarding the actual mAP achieved, the differences observed in Table 3 are less impressive. The best performance was achieved by Yolov8 at 0.543, followed closely by Yolov11 at 0.541 and finally RT-Detr at 0.517. Its counterpart mAP0.50 gave similar results, with Yolov8 achieving 0.781, Yolov11 0.773 and RT-Detr 0.759 and the differences are still minimal. The individual precision in each dataset gave more diverse outcomes. More specifically, inFLIR-ADAS and Corsican Yolov8 took first place, inHIT-UAV Yolov11 won, and finally inCorsican fire RT-Detr had the best performance.

At first glance, the difference in the best mAP or mAP0.5 for each model is minimal, especially between the two Yolos which have a difference of 0.002. On the second-level analysis, when the processing ability of each model is included, we can observe that Yolov8 can process 37 fps, Yolov11 25 frames and RT-Detr 40 frames at the same time. As can be observed, the order is reversed in the processing speed, with first place going to RT-Detr. Thus, in cases where the most important aspect is delivering a quick detection system, the inclusion of RT-Detr might be the best option.

**Figure 1:**
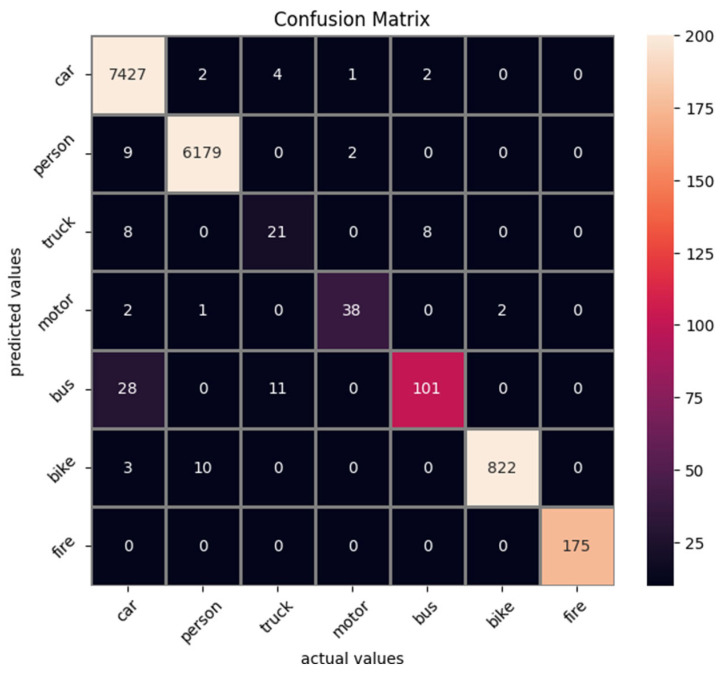
Confusion Matrix of the detected objects vs. the actual objects. The model utilized is the best performing Yolov8 896 × 896 model.

Attempting a more scrutinized analysis per class, we present the best mAP performance for each model and its performance over each class in Table 4. First of all, as is expected, the average precision is quite diverse for specific classes. The lowest performing class is the Truck one, which can be attributed to a low instance number as well as visual proximity with the Bus class especially in the thermal spectrum (in the visual one, there is also the advantage of clearer images and color information). To further elaborate for the misclassification of each class, a Confusion Matrix has been produced, as displayed in Figure 1. The model which was selected to produce the Confusion Matrix is the best performing one, Yolov8 medium at 896 image resolution. It can be seen that indeed the majority of the Truck objects belong to the Bus class, followed by the Car class. The threshold for validating a detection is set to 0.02 (under this threshold, the detection is dismissed), and the threshold for assigning to a ground truth bounding box is an IoU of 0.5 (an IoU value less than this value considers this specific detection to be a false positive). An interesting part is that Yolov11 had the most first places with 5, but Yolov8 had the best overall mAP. Yolov8 and RT-Detr had one first place each. As is obvious from the classes, at least some of them can be combined to improve performance. 

In order to mitigate the imbalance of the dataset, we have attempted certain methods to examine if any of them could improve the performance in the mis-performing classes as well as the overall model performance. In the following table, Table 5, the results of applying certain mitigation methods are presented. The best performing model is chosen, Yolov8, to apply the methods on. Yolov8 medium at 896 image resolution achieves 0.543 mAP, and Truck 0.283 AP. None of the methods achieve higher mAP, with only the weighted classes, in which the Truck was assigned a weight of 20 while all other classes had 1, achieved the same mAP. Nevertheless, even in this case, the Truck mAP was less than the original one, 0.233 vs. 0.283. Thus, we could not encounter any fruitful mitigation method for the poor performance of the Truck class.

Finally, when analyzing the per individual dataset performance, we observe a similar trend with the combined dataset performance. Of the four datasets, the first two places are taken by Yolov8 and two by Yolov11 for the mAP metric. When mAP50 is utilized as a metric, the general image does not change since again the top spots are split between the two Yolos. As a conclusion, Yolos share a similar partner, and thus, their performance is quite close in all four datasets while RT-Detr somehow deviates from this pattern.

The comparison between the CNN-based detectors (Yolovs) and the transformer ones (RT-Detr v2) indicated a superiority of the former which might seem counterintuitive. We suspect this behavior to be boosted by the small dataset size since transformers tend to be more reliable to large dataset availability [63]. Since the datasets being utilized for this paper are relatively small, this could be the reason for performing worse than the CNN-based counterparts. A solution to this drawback is to take advantage of the ability of the transformers to extract generic features from their (typically large) training set and later be successfully fine-tuned in a smaller dataset. This approach often proves to be fruitful because the features transformers learn tend to be generic, and thus, highly robust to transfer to different tasks [64]. The transformer utilized in these experiments was trained on ImageNet and thus, the features learned should be considered generic enough. A possible explanation for its under-performance could be that the model was trained through exploiting transfer learning with all of its layers unfrozen. Possibly, a more conservative approach would have kept more generic features intact and produced better results. 

In the following Figure 2, Figure 3, Figure 4, Figure 5, Figure 6, Figure 7, Figure 8, Figure 9, Figure 10 and Figure 11, detection examples are presented to visualize the detection ability of the models. In each example, only the best model’s output is depicted in each dataset (Yolov8 or Yolov11).

**Figure 12:**
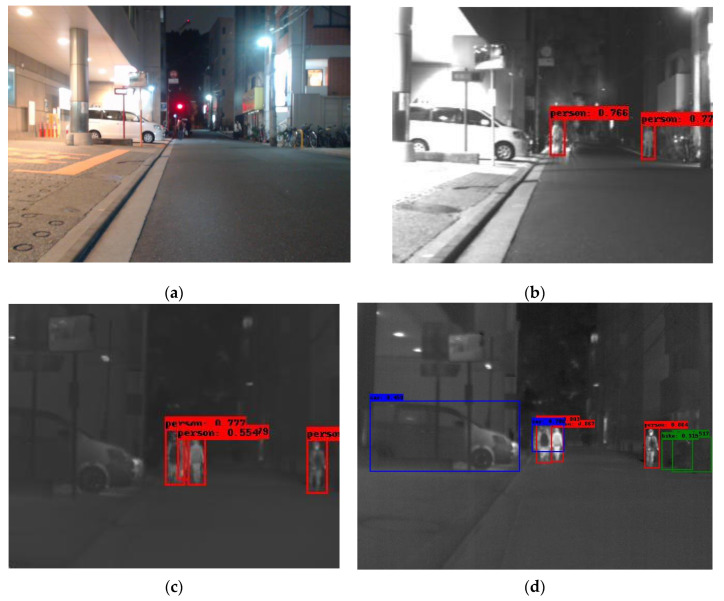
A detection example from the SMOD dataset using Yolov8. (**a**) The original RGB image (shown for object demonstration); (**b**) the detected objects on the nir image are shown as overlay bounding boxes; (**c**) the detected objects on the mir image are shown as overlay bounding boxes; (**d**) the detected objects on the fir image are shown as overlay bounding boxes.

**Figure 13:**
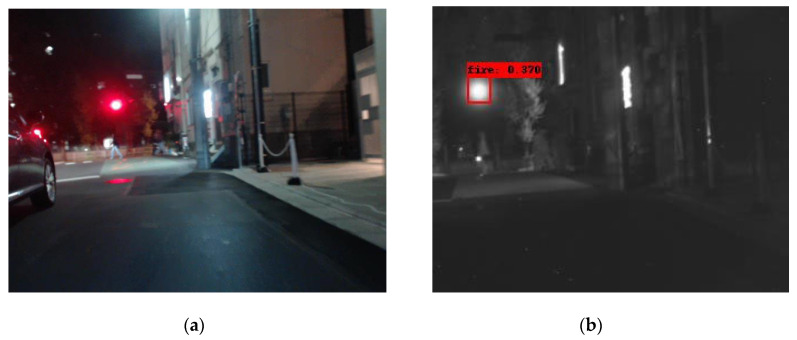

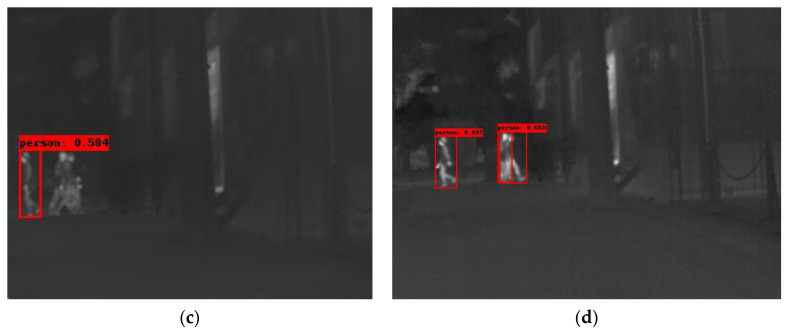
A second detection example from the SMOD dataset using Yolov8. (**a**) The original RGB image (shown for object demonstration); (**b**) the detected objects on the nir image depicted as overlay bounding boxes; (**c**) the detected objects on the mir image depicted as overlay bounding boxes; (**d**) the detected objects on the fir image depicted as overlay bounding boxes.

To further examine the models’ generalization ability, we have selected a subset of the SMOD multi-modal dataset [65]. This dataset contains images from four modalities: RGB, fir (far IR), mir (middle IR) and nir (Near IR). We selected the first 500 images and performed inference on this subset. The four modalities do not contain images in the same resolution and most importantly the same field of view, which might have an impact on the model’s performance. The latter fact is revealed in the ground truth of the objects annotated in each modality. The RGB subset contains 458 images with objects in it, the fir subset 461, the mir subset 436 and the nir 447. It is obvious that RGB and fir have the larger field of view, while mir and nir images cover a much smaller area. Another observation is that nir images are visually closer to the visual spectrum than to fir ones. This explains the poor performance of our model on it, since our training did not contain any image from the visual spectrum (although the Corsican dataset is captured in the nir band). The following figures, Figure 12 and Figure 13, contain examples of this inference on SMOD. As a conclusion of this inference experiment, we can infer the following: 

(a) The model’s modality is crucial for its detection ability (nir modality performs worse than the fir one for example).

(b) The visual differences in the modality trained versus the one being evaluated against can cause false positives (in the first nir image the traffic light is detected as a fire instance) of false negatives.

(c) To provide the model with robust generalized detection abilities, a diverse and possibly multi-modal thermal dataset would be beneficiary.

## 6. Conclusions

In this work, comprehensive research into the ability of IR/thermal object detection models to fulfill their role as the backbone for the IR/thermal security detection system has been performed. The models included to participate were state-of-the-art examples and were chosen for their stellar performance. Additionally, a second requirement for the models was their efficiency: every model examined can provide a real-time processing speed for image resolutions up to 1024 pixels even when examined in middle-end GPU cards. 

For the research activity, a compilation of thermal/IR publicly available datasets was utilized. The compiled dataset underwent filtering for the irrelevant classes—which were ruled out—and manual annotation for the object instances present but not annotated. This led to an unbalanced dataset with which we attempted to cover the requirement for detections. The performance of each model provided efficient operation, yet their performance in detection ability was lower than expected with the better mAP reaching 0.544. This fact is mainly attributed to the lack of a larger dataset, which would cover more diverse object instances, capturing angles and distances. Through the experiments, it was shown that larger image resolution or model power does not guarantee better overall performance. The latter fact in combination with the decrease in model efficiency as its processing power increases, tends to promote medium models and resolutions as the best options. Nevertheless, this paper provided evidence that a security system can benefit by incorporating IR/thermal detection in its set of tools to further improve its detection ability and armor it against undetected threats.

## Figures and Tables

**Figure 2:**
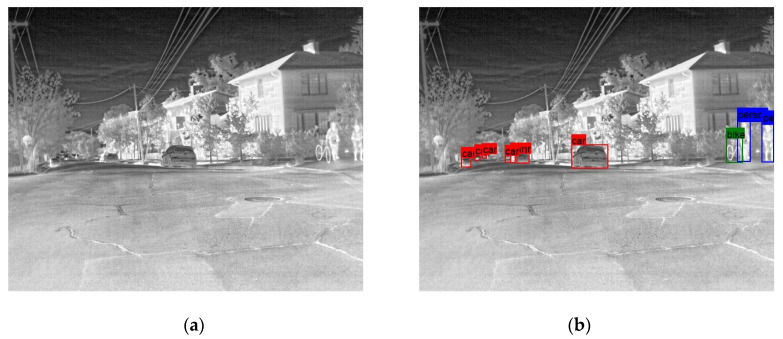
Detection example from the FLIR-ADAS dataset using Yolov8. (**a**) The original image from the dataset; (**b**) the detected objects being annotated as overlayed bounding boxes.

**Figure 3:**
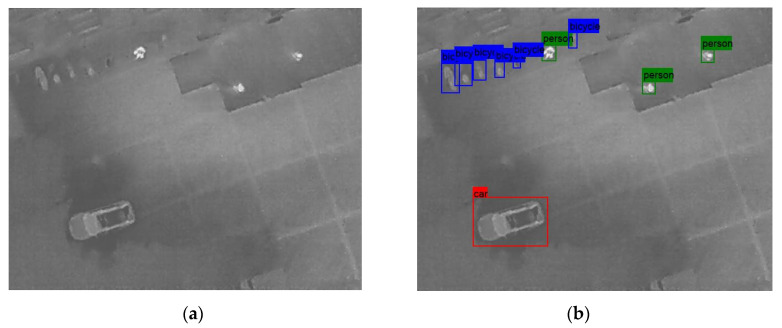
Detection example from the HIT-UAV dataset using Yolov11. (**a**) The original image from the dataset; (**b**) the detected objects being annotated as overlayed bounding boxes.

**Figure 4:**
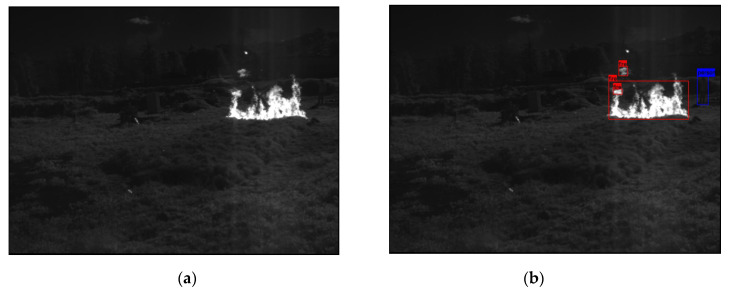
Detection example from the Corsican Fire dataset using Yolov8. (**a**) The original image from the dataset; (**b**) the detected objects being annotated as overlayed bounding boxes.

**Figure 5:**
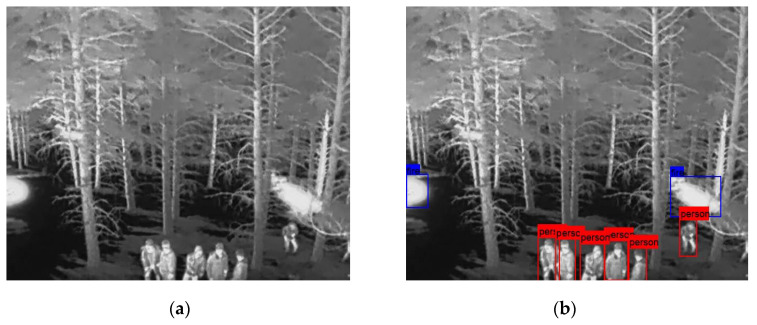
Detection example from the Flame dataset using Yolov11. (**a**) The original image from the dataset; (**b**) the detected objects being annotated as overlayed bounding boxes.

**Figure 6:**
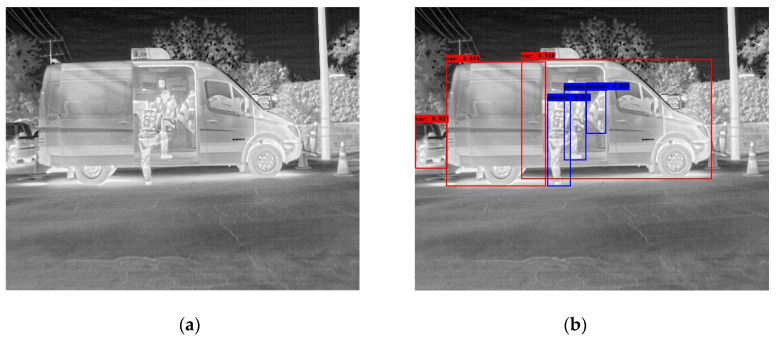
Detection example containing errors from the FLIR-ADAS dataset using Yolov8. (**a**) The original image from the dataset; (**b**) the detected objects being annotated as overlayed bounding boxes. The car (van) is detected as two distinct instances.

**Figure 7:**
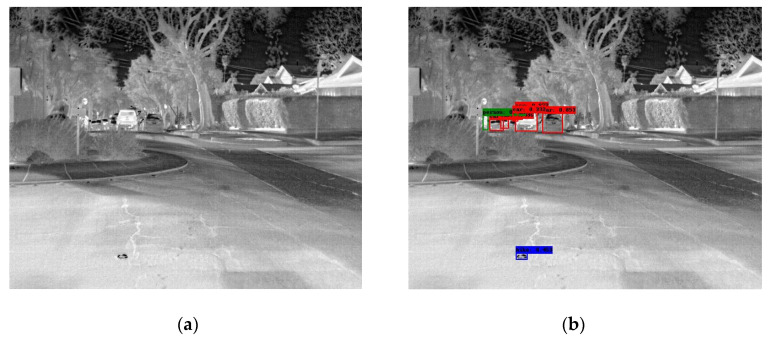
Detection example containing errors from the FLIR-ADAS dataset using Yolov8. (**a**) The original image from the dataset; (**b**) the detected objects being annotated as overlayed bounding boxes. A false positive bike being detected.

**Figure 8:**
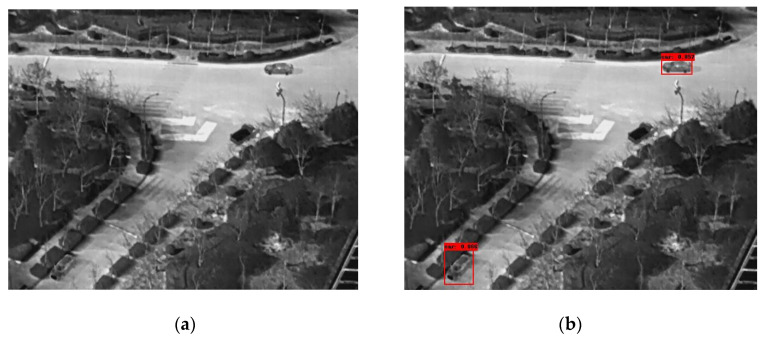
Detection example containing errors from the HIT-UAV dataset using Yolov8. (**a**) The original image from the dataset; (**b**) the detected objects being annotated as overlayed bounding boxes. A car is not being detected (false negative).

**Figure 9:**
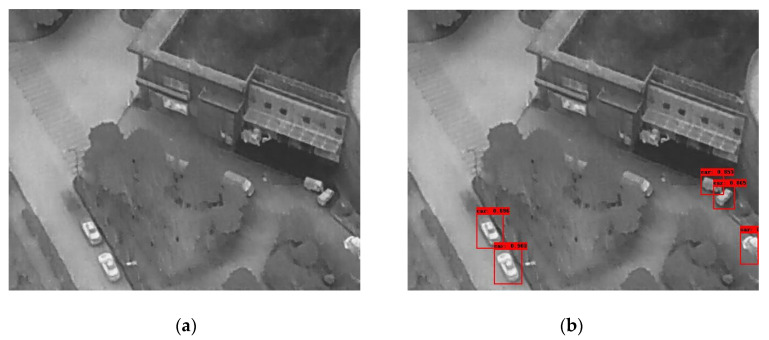
A second detection example containing errors from the HIT-UAV dataset using Yolov8. (**a**) The original image from the dataset; (**b**) the detected objects being annotated as overlayed bounding boxes. A car is not being detected (false negative).

**Figure 10:**
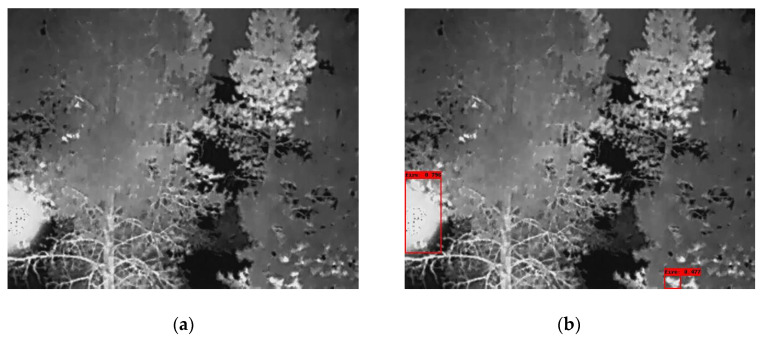
Detection example containing errors from the Flame v1 dataset using Yolov8. (**a**) The original image from the dataset; (**b**) the detected objects being annotated as overlayed bounding boxes. A non-existent fire instance is being detected (false positive).

**Figure 11:**
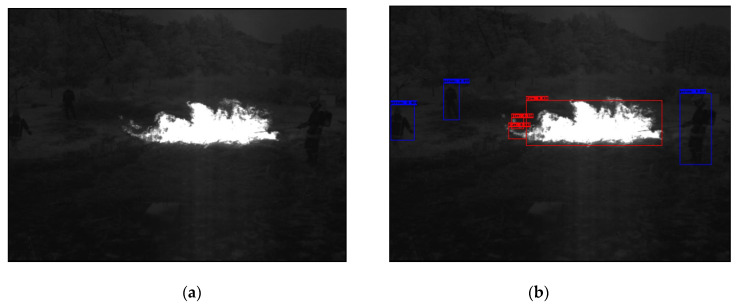
Detection example containing errors from the Corsican Fire dataset using Yolov8. (**a**) The original image from the dataset; (**b**) the detected objects being annotated as overlayed bounding boxes. A fire instance is being detected thrice (two false negatives).

**Table 1:** Number of classes in the datasets used.

Class	Dataset				
	FLIR-ADAS [8]	HIT-UAV [54]	Corsican Fire [55]	Flame [56]	Combined
Person	✓ (50,478)	✓ (12,312)	∗ (1107)	∗ (94)	63,991 (39.0%)
Car	✓ (73,623)	✓ (7311)	∗ (147)	n/a	81,081 (49.4%)
Bike	✓ (7237)	✓ (4980)	n/a	n/a	12,217 (7.4%)
Motorcycle	✓ (1116)	n/a	n/a	n/a	1116 (0.7%)
Bus	✓ (2245)	n/a	n/a	n/a	2245 (1.4%)
Train	✗ (5)	n/a	n/a	n/a	n/a
Truck	✓ (829)	n/a	n/a	n/a	829 (0.5%)
Traffic light	✗ (16,198)	n/a	n/a	n/a	n/a
Fire Hydrant	✗ (1095)	n/a	n/a	n/a	n/a
Street Sign	✗ (20,770)	n/a	n/a	n/a	n/a
Dog	✗ (4)	n/a	n/a	n/a	n/a
Deer	✗ (8)	n/a	n/a	n/a	n/a
Skateboard	✗ (29)	n/a	n/a	n/a	n/a
Stroller	✗ (15)	n/a	n/a	n/a	n/a
Scooter	✗ (15)	n/a	n/a	n/a	n/a
Other Vehicle	✗ (1373)	✗ (148)	n/a	n/a	n/a
Don’t Care	n/a	✗ (148)	n/a	n/a	n/a
Fire	n/a	n/a	✓ (1021)	✓ (1689)	2710 (1.7%)
Sum	135,528	24,603	2275	1783	164,189

Regarding the symbols used in Table 1: ✓ means class was found in the original dataset and also in the final annotation. ✗ the class was included in the original dataset but was excluded from the dataset utilized in this work. N/a (not available) means the class is not present in the dataset at all and the ∗ annotation for this class was missing in the original but added to the final dataset. (xxx) represents the number of object instances inside the dataset.

**Table 2:** Efficiency results of the models.

Model	Variation	Image Resolution	Fps	Parameters (M)	GFlops
Yolov8	small	640 × 640	80	11.1	28.8
		768 × 768	66	11.1	41.5
		896 × 896	51	11.1	56.5
		1024 × 1024	42	11.1	73.8
	medium	640 × 640	56	25.9	79.3
		768 × 768	45	25.9	114.2
		896 × 896	37	25.9	155.5
		1024 × 1024	30	25.9	203.1
Yolo11	small	640 × 640	56	9.4	21.7
		768 × 768	48	9.4	31.3
		896 × 896	43	9.4	42.6
		1024 × 1024	35	9.4	55.6
	medium	640 × 640	48	20.1	68.5
		768 × 768	38	20.1	98.7
		896 × 896	31	20.1	134.3
		1024 × 1024	25	20.1	175.4
RT-Detr v2	r18	640 × 640	73	20.0	61.1
		768 × 768	63	20.0	87.1
		896 × 896	58	20.0	117.8
		1024 × 1024	54	20.0	153.4
	r34	640 × 640	57	31.3	93.2
		768 × 768	48	31.3	132.9
		896 × 896	44	31.3	180.0
		1024 × 1024	40	31.3	234.3

**Table 3:** Effectiveness results of the models (mAP/mAP^50^).

Model	Variation	Image Resol.	FLIR-ADAS v2	HIT-UAV	Corsican	Flame	Combined
Yolov8	small	640 × 640	0.429/0.647	0.642/0.946	0.805/0.970	0.579/0.866	0.501/0.749
		768 × 768	0.435/0.665	0.646/0.946	0.827/0.974	0.614/0.901	0.509/0.751
		896 × 896	0.454/0.676	0.650/0.948	0.826/0.973	0.585/0.873	0.513/0.757
		1024 × 1024	0.452/0.667	0.649/0.949	0.822/0.974	0.586/0.870	0.518/0.753
	medium	640 × 640	0.460/0.679	0.643/0.947	0.826/0.979	0.604/0.878	0.525/0.762
		768 × 768	0.461/0.680	0.645/0.944	0.823/0.972	0.624/0.872	0.532/0.765
		896 × 896	0.475/0.692	0.653/0.948	0.811/0.969	0.623/0.907	0.543/0.781
		1024 × 1024	0.470/0.689	0.653/0.947	0.820/0.965	0.597/0.849	0.538/0.774
Yolo11	small	640 × 640	0.425/0.664	0.642/0.948	0.819/0.969	0.606/0.926	0.529/0.769
		768 × 768	0.437/0.673	0.651/0.946	0.808/0.974	0.600/0.891	0.520/0.757
		896 × 896	0.447/0.668	0.652/0.950	0.820/0.974	0.598/0.901	0.514/0.754
		1024 × 1024	0.448/0.670	0.653/0.949	0.813/0.965	0.591/0.886	0.499/0.740
	medium	640 × 640	0.451/0.681	0.649/0.947	0.817/0.970	0.630/0.903	0.525/0.770
		768 × 768	0.463/0.680	0.653/0.953	0.821/0.974	0.628/0.902	0.539/0.772
		896 × 896	0.470/0.689	0.657/0.950	0.819/0.969	0.622/0.885	0.538/0.773
		1024 × 1024	0.468/0.684	0.653/0.953	0.818/0.972	0.608/0.885	0.541/0.773
RT-Detr v2	r18	640 × 640	0.425/0.648	0.622/0.948	0.808/0.947	0.587/0.915	0.494/0.739
		768 × 768	0.437/0.653	0.627/0.945	0.803/0.953	0.605/0.916	0.509/0.750
		896 × 896	0.454/0.676	0.629/0.947	0.799/0.961	0.595/0.882	0.516/0.758
		1024 × 1024	0.446/0.661	0.632/0.947	0.803/0.965	0.604/0.911	0.503/0.735
	r34	640 × 640	0.450/0.684	0.625/0.950	0.779/0.950	0.630/0.902	0.502/0.759
		768 × 768	0.440/0.682	0.620/0.949	0.789/0.954	0.605/0.871	0.509/0.752
		896 × 896	0.445/0.667	0.608/0.938	0.790/0.953	0.601/0.879	0.517/0.758
		1024 × 1024	0.445/0.668	0.594/0.926	0.791/0.953	0.651/0.888	0.514/0.755

**Table 4:** Per class mAP performance of the models.

Model	All	Car	Person	Truck	Motorcycle	Bus	Bicycle	Fire
Yolov8	0.543	0.693	0.557	0.283	0.443	0.531	0.584	0.707
Yolo11	0.541	0.703	0.566	0.214	0.479	0.532	0.587	0.702
RT-Detr	0.517	0.674	0.536	0.189	0.443	0.502	0.552	0.720

**Table 5:** Weighted training for the Yolov8 model.

Model	All	Car	Person	Truck	Motorcycle	Bus	Bicycle	Fire
Yolov8m 896	0.543	0.693	0.557	0.283	0.443	0.531	0.584	0.707
Yolov8m 1024	0.538	0.701	0.564	0.229	0.460	0.521	0.583	0.711
Yolov8m weighted dataloader 896 × 896	0.526	0.677	0.544	0.193	0.472	0.521	0.573	0.703
Yolov8m weighted dataloader 1024 × 1024	0.519	0.689	0.552	0.218	0.407	0.507	0.564	0.700
Yolov8m weighted classes Truckx20	0.543	0.697	0.558	0.233	0.465	0.534	0.583	0.732

## Data Availability

Data generated during this study can be acquired upon request to the authors.

## Abbreviations

The following abbreviations are used in this manuscript:

IR	Infrared
RGB	Red Green Blue
RCNN	Region-based Convolutional Neural Network
GB	Giga Bytes
CNN	Convolutional Neural Network
GPU	Graphics Processing Unit
HSV	Hue Saturation Value
